# Probiotic cocktails accelerate baicalin metabolism in the ileum to modulate intestinal health in broiler chickens

**DOI:** 10.1186/s40104-023-00974-6

**Published:** 2024-02-18

**Authors:** Mingkun Gao, Chaoyong Liao, Jianyang Fu, Zhonghua Ning, Zengpeng Lv, Yuming Guo

**Affiliations:** 1 State Key Laboratory of Animal Nutrition and Feeding, College of Animal Science and Technology, China Agricultural University, Beijing, 100193 China; 2 National Engineering Laboratory for Animal Breeding, College of Animal Science and Technology, China Agricultural University, Beijing, 100193 China

**Keywords:** Baicalin, Gut health, Ileal microbiota, Probiotic cocktails, Synergistic effects

## Abstract

**Background:**

Baicalin and probiotic cocktails are promising feed additives with broad application prospects. While probiotic cocktails are known to enhance intestinal health, the potential synergistic impact of combining baicalin with probiotic cocktails on the gut health of broiler chickens remains largely unexplored. Therefore, this study aims to investigate the influence of the combined administration of baicalin and probiotic cocktails on the composition of ileal and cecal microbiota in broiler chickens to elucidate the underlying mechanisms responsible for the health-promoting effects.

**Results:**

A total of 320 1-day-old male Arbor Acres broilers were divided into 4 groups, each with 8 replicates of 10 chicks per replicate. Over a period of 42 d, the birds were fed a basal diet or the same diet supplemented with 37.5 g/t baicalin (BC), 1,000 g/t probiotic cocktails (PC), or a combination of both BC (37.5 g/t) and PC (1,000 g/t). The results demonstrated that BC + PC exhibited positive synergistic effects, enhancing intestinal morphology, immune function, and barrier function. This was evidenced by increased VH/CD ratio, sIgA levels, and upregulated expression of occludin and claudin-1 (*P* < 0.05). 16S rRNA analysis indicated that PC potentiated the effects of BC, particularly in the ileum, where BC + PC significantly increased the α-diversity of the ileal microbiota, altered its β-diversity, and increased the relative abundance of *Flavonifractor* (*P* < 0.05), a flavonoid-metabolizing bacterium. Furthermore, *Flavonifractor* positively correlated with chicken ileum crypt depth (*P* < 0.05). While BC + PC had a limited effect on cecal microbiota structure, the PC group had a very similar microbial composition to BC + PC, suggesting that the effect of PC at the distal end of the gut overshadowed those of BC.

**Conclusions:**

We demonstrated the synergistic enhancement of gut health regulation in broiler chickens by combining baicalin and probiotic cocktails. Probiotic cocktails enhanced the effects of baicalin and accelerated its metabolism in the ileum, thereby influencing the ileal microbiota structure. This study elucidates the interaction mechanism between probiotic cocktails and plant extract additives within the host microbiota. These findings provide compelling evidence for the future development of feed additive combinations.

## Introduction

Maintaining optimal intestinal health in poultry is crucial to maximizing growth performance and preventing economic losses on poultry farms. To effectively address intestinal health issues, the combination of functional feed additives has gained attention. In particular, the integration of plant extracts with probiotics has emerged as a promising approach. Recent evidence suggest that this combination enhances the medicinal effects of plant extracts by leveraging the catabolic properties of probiotics [1]. Plant extract can function as prebiotics by fostering probiotic proliferation, enhancing their activity, and improving their survival and colonization in the animal intestine [2, 3]. These findings open new avenues for exploring the synergistic use of plant extracts and probiotics to improve animal gut health.

Baicalin is a type of flavonoid extracted from *Scutellaria baicalensis*, which is known for its multiple beneficial effects, including antibacterial, antioxidant, and immunoregulatory properties [4]. The active participation of the gut microbiota is crucial for the metabolism of baicalin into biologically active compounds such as baicalein, baicalin-6-glucuronide, and baicalin-7-glucuronide [5]. Moreover, baicalin can bind directly to intestinal epithelial cells as a protective barrier against bacterial invasion. This intricate interplay with the gut microbiota plays a critical role in maintaining host intestinal health, particularly in preserving the integrity of critical physical barriers such as tight junction proteins like ZO-1 and claudin-1 [6]. In addition, baicalin helps reduce inflammation in broiler chickens by inhibiting the TLR4-mediated NF-κB pathway [7], thereby strengthening the intestinal immune barrier.

Probiotics play a critical role in balancing gut microbes and mitigating the effects of toxic metabolites, thereby improving gut health. Widely recognized for improving production performance, feed additives mainly include *Bacillus subtilis*, *Lactobacillus plantarum*, and *Saccharomyces cerevisiae* [8–10]. *Lactobacillus* produces bacteriocins, lactic acid, and butyric acid, which provide energy to intestinal epithelial cells [11]. Combining *Bacillus subtilis* with *Lactobacillus plantarum* improves broiler intestinal barrier function [12, 13], while *Saccharomyces cerevisiae* enhances the probiotic effect of *Bacillus subtilis* and *Lactobacillus plantarum* [14, 15]. Studies show that feeding flavonoids significantly increases the relative abundance of *Bacillus* and *Lactobacillus* in the gut microbiota, suggesting their involvement in flavonoid metabolism and intestinal health [16]. However, research on baicalin in poultry has primarily focused on its antioxidant and anti-inflammatory effects in models of inflammation or stress. The hypothesis of this study posits that the synergistic combination of baicalin and probiotic cocktails will result in the establishment of a more favorable gut microenvironment, thereby leading to enhanced intestinal health in broiler chickens.

This study explores the synergistic effects of baicalin and probiotic cocktails on intestinal health in broiler chickens. Analysis of the microbial composition analysis in the cecum and ileum will elucidate the effect of combined probiotics on baicalin metabolism. It will also shed light on the relationship between baicalin, probiotic cocktails, and the host intestine and uncover their underlying mechanisms.

## Materials and methods

### Ethics statement

All animal procedures were performed by the Guidelines for Care and Use of Laboratory Animals of China Agricultural University, and the experimental protocol was approved by the Animal Care and Use Committee of China Agricultural University (Approval No. Aw61902202-1-8).

### Materials

Arbor Acre (AA) broilers, healthy 1-day-old males, were obtained from Beijing Huadu Yukou commodity generation. Baicalin (Purity > 85%) was sourced from Beijing Centre Biology Co., Ltd. (Beijing, China). The probiotic cocktails were purchased from Shandong Sukahan Bio-Technology Co., Ltd. (Shandong, China), mainly containing *Bacillus subtilis* (≥ 5.0 × 10^8^ CFU/g), *Lactobacillus plantarum* (≥ 3.0 × 10^7^ CFU/g), and *Saccharomyces cerevisiae* (≥ 5.0 × 10^8^ CFU/g).

### Experimental design and diets

A total of 320 AA broilers with a mean body weight of 37.3 ± 0.1 g were randomly allocated into 4 treatment groups, with 8 replicate pens per group, each containing 10 chicks. The experiment comprised the early feed stage from 1 to 21 d and the late feed stage from 22 to 42 d. The broilers' diet formula followed the feeding standards for Chinese chickens (NY/T33-2004) [17], as shown in Table 1.

**Table 1:** Composition and nutrient levels of basal diet (air-dry basis)

Ingredients, %	1–21 d	22–42 d
Corn (7.8% CP)	50.85	59.76
Soybean meal (46% CP)	35.90	20.00
Corn gluten meal (51.3% CP)	5.00	10.00
Soybean oil	3.93	3.80
Wheat shorts	-	2.00
Dicalcium phosphate	2.00	1.20
Limestone	1.20	1.85
NaCl	0.35	0.35
Choline chloride (50%)	0.30	0.30
Mineral premix^a^	0.20	0.20
DL-Methionine (98%)	0.21	0.16
L-Lysine monohydrochloride (78%)	0.03	0.35
Vitamin premix^b^	0.03	0.03
Total	100.00	100.00
Nutrient levels		
Metabolizable energy, Mcal/kg	2.99	3.17
Crude protein, %	22.30	20.36
Lysine, %	1.15	1.07
Methionine, %	0.54	0.50
Tryptophan, %	0.27	0.25
Threonine, %	0.82	0.74
Met + Cys, %	0.95	0.86
Calcium, %	1.05	1.01
Available phosphorus, %	0.45	0.40

The phytase activity was 10,000 U/g in the enzyme supplement

^a^The composite mineral supplement provided the following amounts per kilogram of diet: copper 8 mg, zinc 75 mg, iron 80 mg, manganese 100 mg, selenium 0.15 mg, iodine 0.35 mg, and cobalt 0.5 mg

^b^The composite vitamin supplement provided the following amounts per kilogram of diet: vitamin A 12,500 IU, vitamin D_3_ 2,500 IU, vitamin K_3_ 2.65 mg, vitamin B_1_ 2 mg, vitamin B_2_ 6 mg, vitamin B_12_ 0.025 mg, vitamin E 30 IU, biotin 0.0325 mg, folic acid 1.25 mg, pantothenic acid 12 mg, and niacin 50 mg

The control group (CON) received a basic diet, while the baicalin group (BC) received the CON diet supplemented with BC at 37.5 g/t. The probiotic cocktail group (PC) received the CON diet supplemented with the PC at 1,000 g/t. The BC + PC group received the CON diet supplemented with baicalin (37.5 g/t) and the probiotic cocktail (1,000 g/t), with the dietary supplementation commencing from the first day of chick rearing. Each replicate was housed in a separate pen, and the broilers were kept in enclosed rooms with 2-layer galvanized iron wire cages and ventilation provided by exhaust fans. The room temperature was maintained around 35 °C during the first week and gradually decreased to a constant temperature of 25 °C. Relative humidity was maintained between 65% and 70%. Throughout the experimental period, all chicks had ad libitum access to food and water, and artificial light was provided with a 23-h lighting and 1-h darkness schedule using fluorescent lights.

### Sampling procedure

At 21 d and 42 d, chickens were weighed by replication, and the feed consumption was recorded by replication. The average body weight (BW), body weight gain (BWG), feed intake (FI), and feed conversion ratio (FCR) were calculated for the periods from 1 to 21 d, 21 to 42 d, and 1 to 42 d. On 21 d and 42 d, one chick of average BW was randomly selected from each cage. The chickens were killed by jugular exsanguination. Organs (thymus, spleen, liver, and bursa of Fabricius) were removed and weighed. We then carefully collected the ileum and cecum. Sections of 1 cm were cut off from the middle of the ileum. The ileum sections were then fixed in 4% paraformaldehyde. The remaining length of the ileum was stored at −80 °C for further analysis. The ileal and cecal chyme of chickens at 21 d were collected and frozen in liquid nitrogen for DNA extraction.

### Determination of serum immunoglobulin

After an 8-h fasting period, blood samples were collected from the wing vein of the birds and then centrifuged at 3,000 r/min for 15 min at 4 °C. The serum was separated and stored at −80 °C for subsequent analysis. The level of IgA in the serum was determined using the Chicken IgA ELISA kit (ml002792, Shanghai Enzyme-linked Biotechnology Co., Ltd., Shanghai, China) following the manufacturer's instructions. The serum IgG levels were also measured using a commercial ELISA kit (ml042771, Shanghai Enzyme-linked Biotechnology Co., Ltd., Shanghai, China).

### The sIgA level of ileum mucosa

After collecting ileal mucosa samples from broilers, the tissue homogenate was prepared using a ratio of mucosal sample to phosphate buffer saline of 1:9. The homogenate was then centrifuged at 3,000 r/min and 4 °C for 15 min to obtain the supernatant for further use. The chicken secretory IgA (sIgA) level was measured using the chicken sIgA ELISA kit (ml002778, Shanghai Enzyme-linked Biotechnology Co., Ltd., Shanghai, China). Following the provided protocol, total protein levels were measured using the BCA protein quantification kit (Cwbio, Beijing, China). The sIgA values were expressed as the level of sIgA per gram of protein.

### Intestinal morphology

After fixation in 4% paraformaldehyde for 24 h, the ileum sections were dehydrated through a series of graded ethanol and xylene and then embedded in paraffin. Transverse 5-μm sections were stained with hematoxylin and eosin. Histomorphological parameters were examined using an Olympus optical microscope and analyzed with Image J Software (version 1.52). The villus height and crypt depth of the slice samples were measured, and the ratio of villus height to crypt depth was calculated. For each slice sample, ten complete and vertical villi were selected. Villus height was measured from the top of the villus to the crypt opening, and crypt depth was measured from the crypt opening to the base of the crypt.

### Gene expression

The ileum samples were collected and immediately placed in RNase-free centrifuge tubes, followed by rapid freezing in liquid nitrogen. Total RNA isolation was performed using 100 mg tissue samples and 1mL Trizol reagent (Invitrogen Life Technologies, Carlsbad, CA, USA), following the method described by a previous study [18]. Total RNA transcription was repeated with the PrimeScript RT reagent kit with gDNA Eraser (Takara, Dalian, China) according to the manufacturer's instructions. For gene expression analysis, RT-PCR was performed with primers listed in Table 2 using the SYBR Premix Ex Taq^TM^ (Takara, Dalian, China) on an Applied Biosystems 7500 Fast Real-Time PCR System (Foster City, CA, USA). The PCR reaction system's total volume was 20 μL.

**Table 2:** Sequences of the oligonucleotide primers used for quantitative real-time PCR

Gene name	Primer sequence(5´→3´)	GenBank accession number
*GAPDH*	F: AGAACATCATCCCAGCGTCC R: CGGCAGGTCAGGTCAACAAC	NM_204305
*IL-1β*	F: TGGGCATCAAGGGCTACA R: CGGCCCACGTAGTAAATGAT	NM_204524.1
*TLR4*	F: GATGCATCCCCAGTCCGTG R: CCAGGGTGGTGTTTGGGATT	NM_001030693
*NF-κB*	F: TGGAGAAGGCTATGCAGCTT R: CATCCTGGACAGCAGTGAGA	NM_205134.1
*IL-10*	F: CGCTGTCACCGCTTCTTCA R: TCCCGTTCTCATCCATCTTCTC	AJ621614
*IFN-γ*	F: AAAGCCGCACATCAAACACA R: GCCATCAGGAAGGTTGTTTTTC	NM_205149.1
*ZO-1*	F: ACAGCTCATCACAGCCTCCT R: TGAAGGGCTTACAGGAATGG	XM_015278981.1
Claudin1	F: GGTATGGCAACAGAGTGGCT R: CAGCCAATGAAGAGGGCTGA	NM_001013611
Occludin	F: AGTTCGACACCGACCTGAAG R: TCCTGGTATTGAGGGCTGTC	NM_205128.1

*IL-1β* Interleukin 1 beta, *TLR4* Toll like receptor 4, *NF-kB* Nuclear factor kappa B, *IL-10* Interleukin 10, *IFN-γ* Interferon gamma

### 16S rDNA sequencing of ileal and cecal bacteria

At the end of 21 d, chyme samples from the distal region of the ileum and cecum were collected. Microbial DNA extraction was performed using the QIAamp Fast DNA Stool Mini kit (Qiagen Company, Dusseldorf, Germany) following the method described by a previous study [18]. Bacterial DNA from the V3-V4 region of the 16S rRNA gene was amplified using universal primers: 338F (5'-ACTCCTACGGGAGGCAGCA-3') and 806R (5'-GGACTACHVGGGTWTCTAAT-3'). PCR products were purified, quantified, and homogenized to create a sequencing library. HiSeq2500 PE250 was used for on-machine sequencing. Beijing Nuohe Zhiyuan Bio-Information Technology Co., Ltd. completed the sequencing analysis. Qiime software (Qiime2-2019.7, Nature Biotechnology) generated species abundance tables at different taxonomic levels and analyzed alpha diversity. The unweighted pair-group method with arithmetic means (UPGMA) was calculated using Quantitative Insights Into Microbial Ecology (version 1.7.0; http://qiime.org/scripts/split_libraries_fastq.html). Additionally, LEfSe analysis was performed to identify biomarkers with statistical differences between groups based on the LDA value. Principal coordinate analysis (PCoA) diagrams were drawn using R software (Version 2.15.3).

### Statistical analysis

The data were analyzed using the conventional linear model program in SPSS 26.0 software (SPSS Inc., Chicago, IL, USA). The general linear model (GLM) process was used for statistical analysis. Duncan's multiple comparisons were used to compare the effects of BC. In case the interaction between BC and PC was observed, One-way ANOVA and Duncan's multiple comparisons were applied. The Pearson correlation coefficient was used to evaluate the correlation between growth performance, intestinal health indicators, and bacteria with differences identified in this study. A significance level of *P* < 0.05 was considered statistically significant, while 0.05 < *P* < 0.10 showed a trend of difference. GraphPad Prism 9.0 software was used for data visualization.

## Result

### Growth performance

To explore the combined effects of BC and PC on broiler chicken growth performance, 1-d birds were fed diets containing 37.5 g/t BC, 1,000 g/t PC, and a combination of 37.5 g/t BC + 1,000 g/t PC for 42 d. The average body weight, feed intake, and feed conversion ratio of broilers from 1 to 42 d in the BC and PC groups showed no significant effects. Likewise, the BC + PC treatment did not yield reciprocal effects (*P* > 0.05, Table 3). However, from 22 to 42 days of age, the BC + PC treatment demonstrated a significant interaction effect on the feed conversion rate (*P* < 0.05).

**Table 3:** Effects of combined baicalin and probiotic cocktails on the production performance

Items	Group				SEM	Main effect				*P*-value		
						BC		PC				
	CON	BC	PC	BC + PC		-	+	-	+	BC	PC	BC × PC
1–21 d												
AW, g	698	679	706	704	10.1	702	692	689	705	0.415	0.185	0.487
FI, g	985	956	958	956	13.4	971	956	971	957	0.268	0.320	0.317
FCR	1.47	1.45	1.45	1.45	0.01	1.46	1.45	1.46	1.45	0.653	0.581	0.924
22–42 d												
AW, g	2,537	2,533	2,523	2,500	33.6	2,530	2,517	2,535	2,511	0.710	0.491	0.781
FI, g	2,909	2,938	2,911	2,947	27.9	2,910	2,942	2,923	2,929	0.281	0.842	0.894
FCR	1.71^a^	1.66^b^	1.67^a^	1.70^a^	0.01	1.69	1.68	1.69	1.68	0.539	0.925	0.024
1–42 d												
BWG, g	2,488	2,479	2,442	2,448	31.0	2,465	2,464	2,484	2,445	0.978	0.267	0.821
FI, g	3,898	3,895	3,692	3,874	95.2	3,795	3,885	3,897	3,783	0.399	0.288	0.381
FCR	1.57	1.57	1.51	1.58	0.03	1.54	1.58	1.57	1.55	0.345	0.547	0.377

*AW* Average weight, *FI *Feed intake, *BC* Baicalin, *PC *Probiotics cocktail, *FCR *Feed conversion ratio, *SEM* Standard error of the mean (*n* = 8)

^a,b^Means in the same row without the same superscript differ significantly (*P* < 0.05). - means not add, + means add

### Immune organ index

Thymus, spleen, and bursa are vital immune organs in birds, with the bursa being a unique central immune organ. The bursa index is an essential indicator to assess the body's immune status. The BC group significantly reduced the bursa index compared to the CON group (*P* < 0.05, Table 4). Moreover, the BC + PC group exhibited an interactive effect on the bursa index (*P* < 0.01) compared to the CON group. Additionally, the BC + PC group demonstrated a significant interactive effect on the bursa and thymus indexes on 42 d (*P* < 0.01) compared to the CON group.

**Table 4:** Effects of combined baicalin and probiotic cocktails on broiler immune organ index

Items, g/t	Group				SEM	Main effect				*P*-value		
						BC		PC				
	CON	BC	PC	BC + PC		-	+	-	+	BC	PC	BC × PC
21 d												
Spleen	0.96^b^	0.79^b^	1.12^a^	1.23^a^	0.05	1.04	1.01	0.88	1.18	0.591	0.001	0.018
Bursa	3.03^a^	2.23^b^	2.63^b^	2.59^b^	0.11	2.83	2.41	2.63	2.61	0.003	0.850	0.007
Thymus	4.11	3.93	3.84	3.95	0.19	3.98	3.94	4.02	3.89	0.857	0.539	0.463
42 d												
Spleen	1.32	1.26	1.25	1.20	0.06	1.28	1.23	1.29	1.23	0.462	0.332	0.937
Bursa	0.49^a^	0.41^b^	0.45^a^	0.53^a^	0.02	0.47	0.47	0.45	0.49	0.949	0.280	0.014
Thymus	2.97^c^	2.63^b^	2.74^c^	3.38^a^	0.09	2.86	3.00	2.80	3.06	0.176	0.022	< 0.001

*BC* Baicalin, *PC* probiotics cocktail, *SEM* Standard error of the mean (*n* = 8)

^a,b^Means in the same row without the same superscript differ significantly (*P* < 0.05). - means not add, + means add

### Immune and intestinal barrier function

**Fig. 1:**
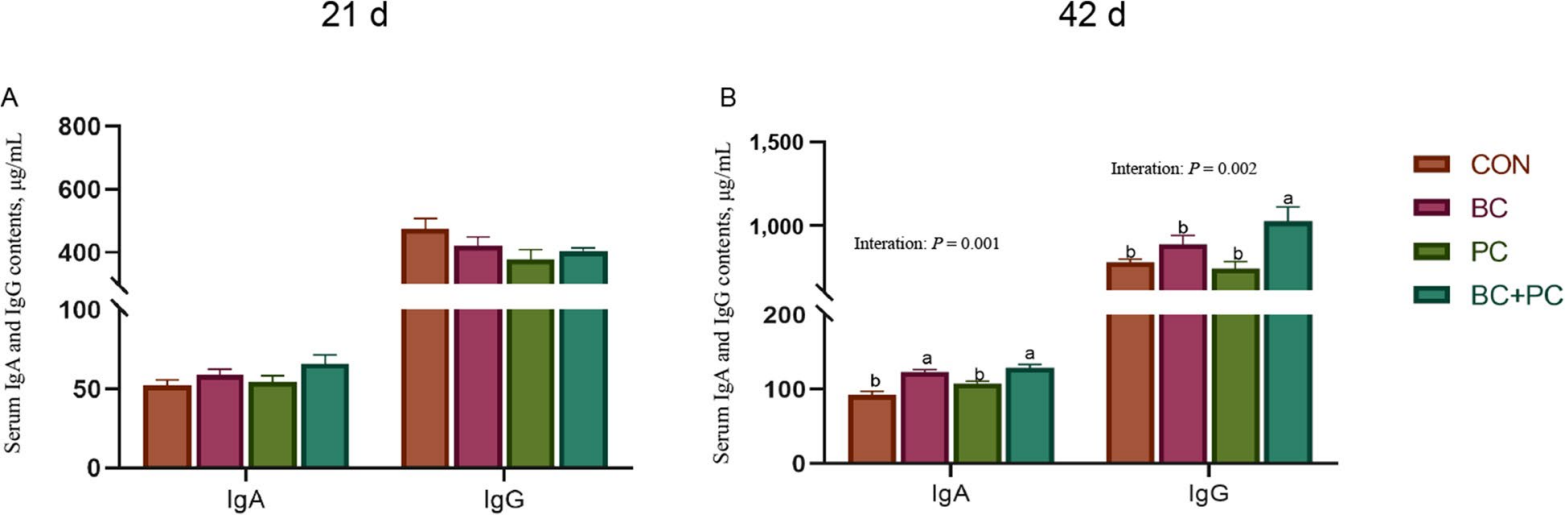
The combined effect of baicalin and probiotics on serum immunoglobulins in broiler chickens. Data are presented as mean ± SEM (*n* = 8). The primary and interaction effects were analyzed using the general linear model (GLM) procedure, with corresponding *P* values displayed over each plot. One-way ANOVA and multiple comparisons were conducted for significant interactive effects. ^a,b^Means denoted by different superscripts within the same row are significantly different (*P* < 0.05)

IgA, IgG, and sIgA play vital roles in broiler chicken immune function, serving as critical antibodies that defend against pathogens and support mucosal immunity. IgA protects the mucosal surfaces, while IgG provides systemic immunity, and sIgA offers dual protection at mucosal sites. The BC and PC groups did not show a significant increase in serum IgG levels at 21 d compared to the CON group (Fig. 1A). However, at 42 d, BC significantly increased serum IgA levels (*P* < 0.01), and the BC + PC group exhibited a positive interaction effect, significantly elevating serum IgA and IgG levels (*P* < 0.05, Fig. 1B).

**Fig. 2:**
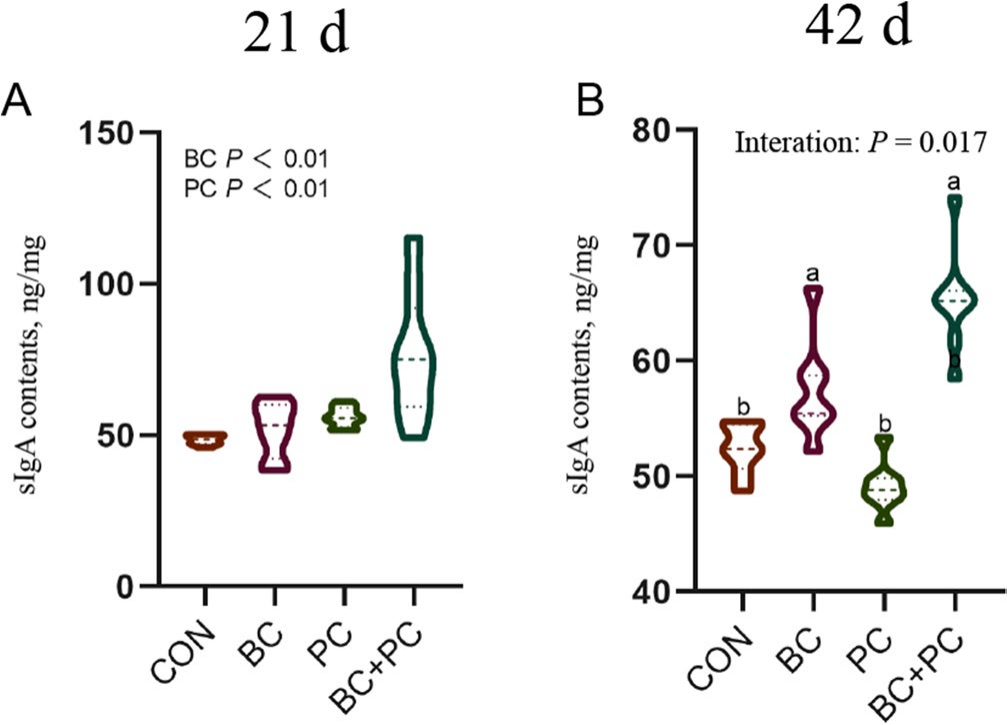
The combined effect of baicalin and probiotics on ileal sIgA in broiler chickens. Data are presented as mean ± SEM (*n* = 8). The main and interaction effects were analyzed using the general linear model (GLM) procedure, with corresponding *P* values displayed over each plot. One-way ANOVA and multiple comparisons were conducted for significant interactive effects. ^a,b^Means denoted by different superscripts within the same row are significantly different (*P* < 0.05)

BC group significantly increased ileal intestinal mucosal sIgA content in 21- and 42-d broiler chickens compared to the control group (*P* < 0.01, Fig. 2A). Additionally, the combined application BC + PC exhibited a positive interactive effect at 42 d, leading to a significant elevation in sIgA content (*P* < 0.05, Fig. 2B).

### Ileal intestinal morphology

**Fig. 3:**
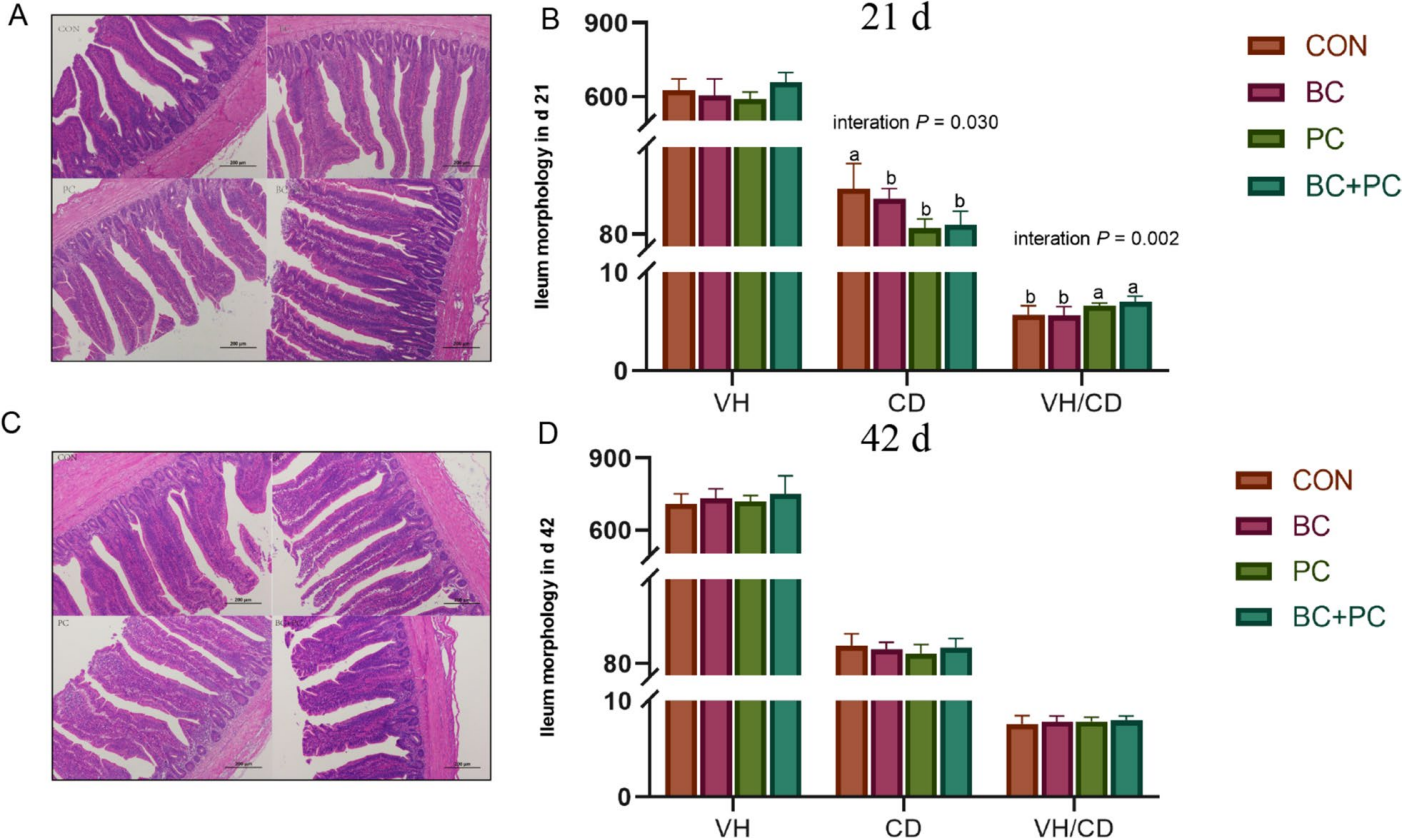
The combined effect of baicalin and probiotics on the ileal histomorphology of 21- and 42-d broiler chickens. Data are presented as mean ± SEM (*n* = 8). The primary and interaction effects were analyzed using the general linear model procedure, with corresponding *P* values displayed over each plot. One-way ANOVA and multiple comparisons were conducted for significant interactive effects. ^a,b^Means denoted by different superscripts within the same row are significantly different (*P* < 0.05)

Ileal morphology is a crucial indicator of broiler chicken intestinal health, where intestinal villi represent nutrient absorption capacity. Both the BC and PC groups significantly reduced the crypt depth of the ileum in 21 d broiler chickens (*P* < 0.05). The BC group significantly increased the VH/CD ratio in 21-d broiler chickens (*P* < 0.01, Fig. 3A and B), and the combined BC + PC treatment demonstrated a positive interactive effect, significantly elevating the VH/CD ratio in 21-d broiler chickens (*P* < 0.01, Fig. 3A and B). However, the BC, PC, and BC + PC treatments did not significantly affect the ileal morphological parameters in 42-d broiler chickens (Fig. 3C and D).

### Microbiota in the ileum and cecum

#### The combined application of BC and PC altered the microbial structure in the ileum

To investigate the combined effects of BC and PC on broiler chicken intestinal microbiota development, 16S rRNA sequencing was performed on the chyme of the ileum and cecum of 21 d chickens. This aimed to explore the impact on gut microbiota and identify the main influential sites.

**Fig. 4:**
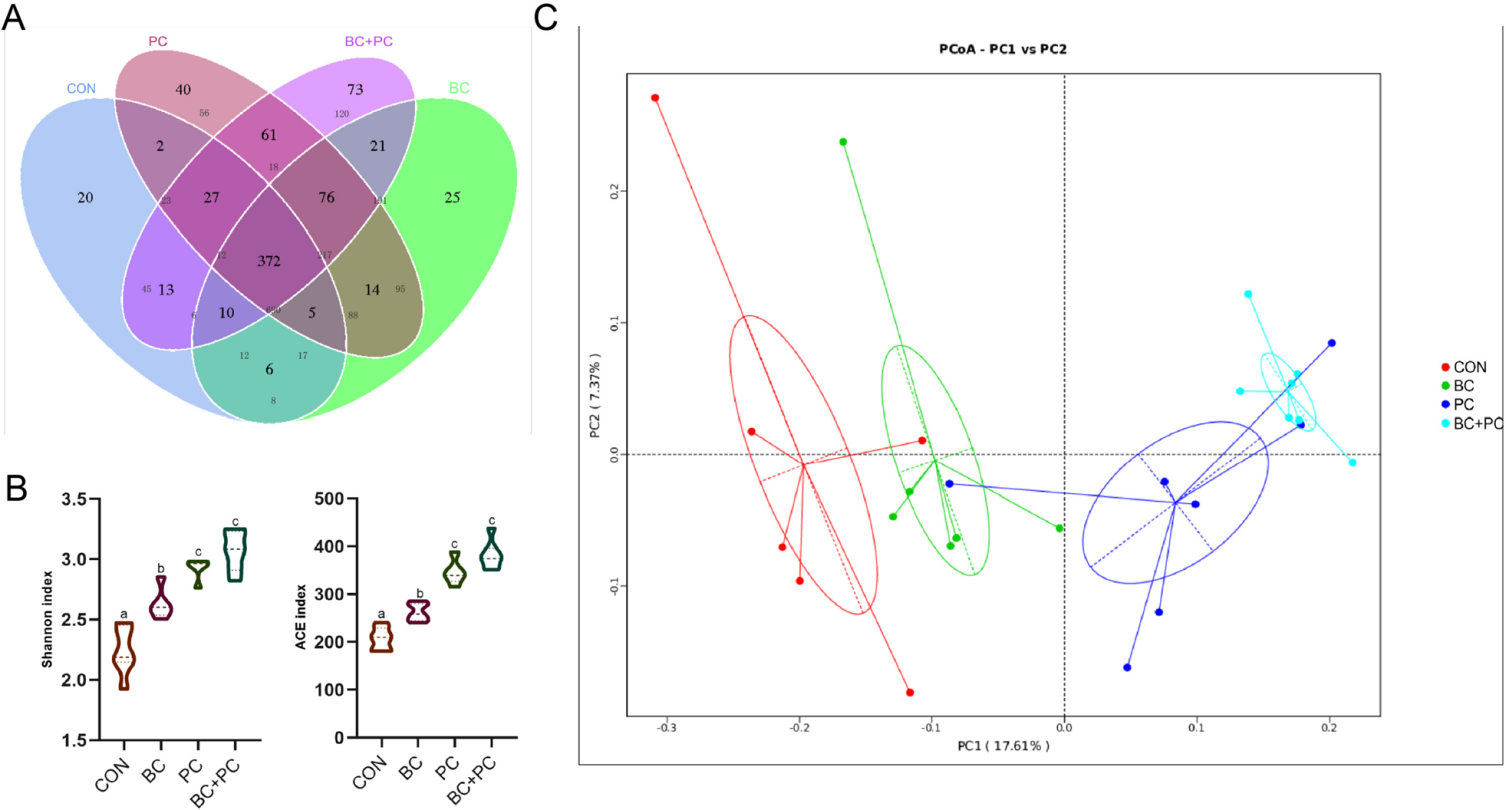
BC + PC changes ileal microbiota community structure. **A** The Venn diagram at the genus level. **B** Microbiome α-diversity was assessed at the genus level in BC, PC, BC + PC, and control groups (*n* = 8). **C** PCoA analysis was performed to evaluate the dissimilarity of ileum microbial community structure (*n* = 8). ^a^^–^^c^Means denoted by different superscripts within the same row are significantly different (*P* < 0.05)

Compared to the CON group, the BC + PC group significantly increased the Shannon and ACE indices of ileal microbiota (*P* < 0.05, Fig. 4B). The Venn diagram revealed 372 shared OTUs across all treatment groups, with 20, 25, 40, and 73 unique OTUs in CON, BC, PC, and BC + PC groups, respectively (Fig. 4A). α-diversity analysis showed that the Shannon and ACE indices were significantly higher in the BC + PC group compared to the control (*P* < 0.05). β-diversity analysis based on unweighted UniFrac distances (Fig. 4C) exhibited distinct separation between the four treatment groups, primarily along the PC2 axis (7.37%) and PC1 axis (17.61%).

**Fig. 5:**
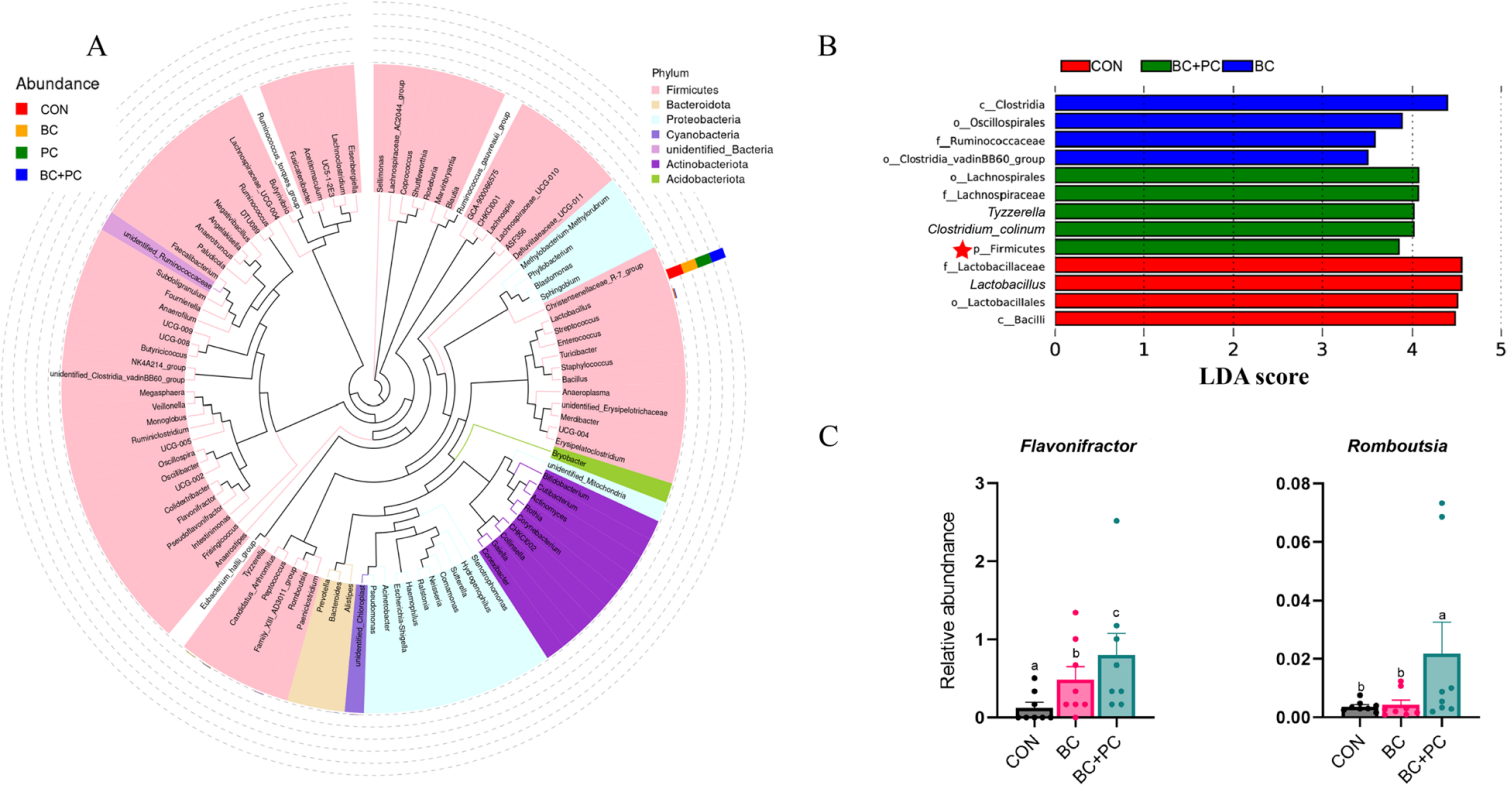
The combined use of PC facilitated the metabolism of BC in the ileal microbiota. **A** Genus-level phylogenetic tree, where the inner circle represents genus-level bacteria, and the outer circle indicates their relative abundances. **B** LEfSe analysis was performed on the CON, BC, PC, and BC + PC groups, with an Linear Discriminant Analysis (LDA) score cutoff of  > 4. **C** Relative abundances of genera were analyzed using ANOVA, and data are presented as mean ± SEM (*n* = 8). ^a^^–^^c^Means with different superscripts within the same row are significantly different (*P* < 0.05)

To investigate the impact of BC + PC on microbial structure, the ileal microbiota was mainly composed of Firmicutes, Bacteroidota, Proteobacteria, and Cyanobacteria at the phylum level, with Lactobacillus being predominant (Fig. 5A). Further examination of the interactive effects of BC + PC on ileal microbial structure using LEfSe (LDA > 4) revealed that the BC group exhibited dominance of Clostridia, Oscillospirales, Ruminococcaceae, and Clostridia_vadinBB60 (Fig. 5B). In contrast, the BC + PC group showed a dominance of Lachnospirales, Lachnospiraceae, *Tyzzerella*, *Clostridium*, and Firmicutes. Notably, the relative abundance of *Flavonifractor*, a flavonoid-metabolizing genus, significantly increased in the BC + PC group compared to the BC group, indicating PC's role in facilitating BC metabolism in the ileum (Fig. 5C). The relative abundance of *Romboutsia* also significantly increased (*P* < 0.05) in the BC + PC group.

### BC + PC increases the modularity of ileum microbial interactions

**Fig. 6:**
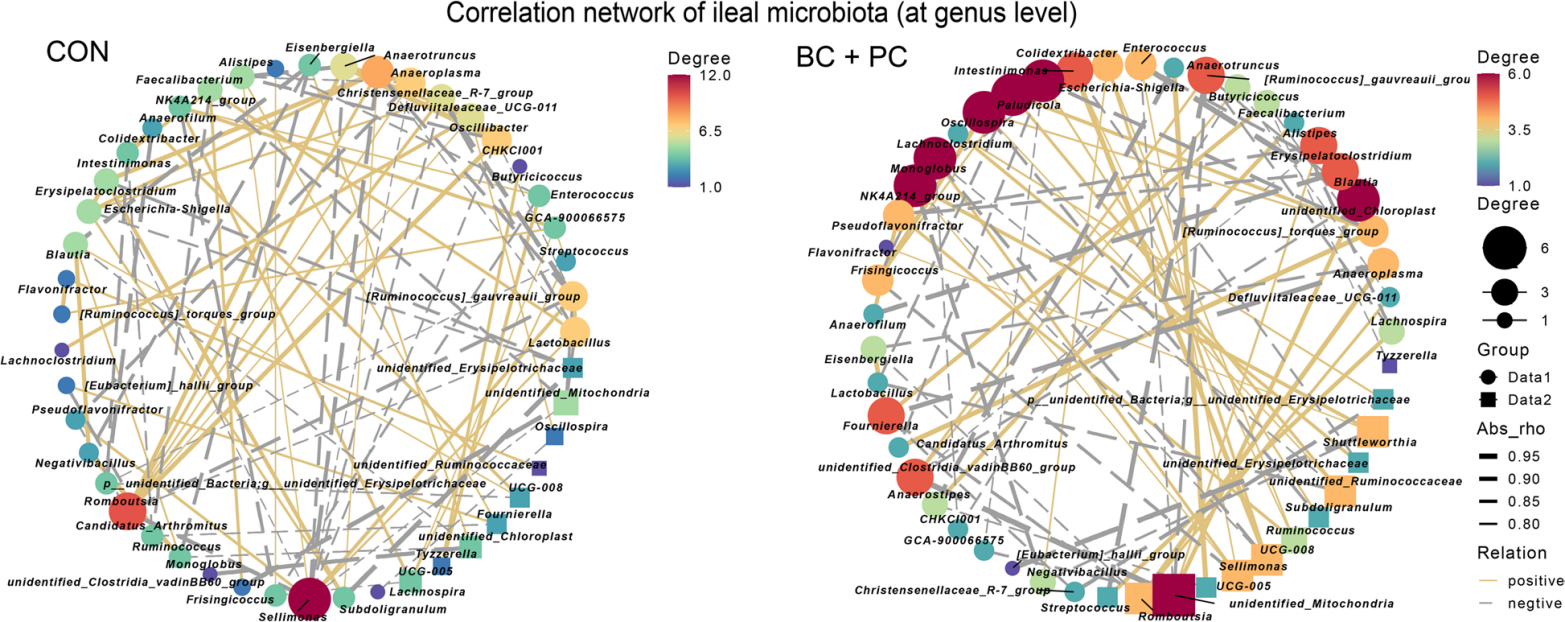
Microbial co-occurrence networks. Co-occurrence networks in the CON and BC + PC groups (*n* = 8). Different colors of nodes indicate different genera. The node size is weighted based on node degree. The golden and silver links represent positive and negative correlations, respectively

To explore the interactive effects of BC and PC on the ileal microbiota regulation, network analysis was conducted separately for the CON and BC + PC groups. This identified unique characteristics of the BC + PC ileal microbiota, with *Romboutsia* and *Sellimonas* being the main positively correlated genera in the CON group (Fig. 6). After using BC + PC, *Romboutsia* and *Lachnoclostridium* promoted microbial development, showing positive correlations with certain short-chain fatty acid-producing bacteria like *Clostridium sporogenes* (Fig. 6).

### Prediction of ileal microbiota functionality

**Fig. 7:**
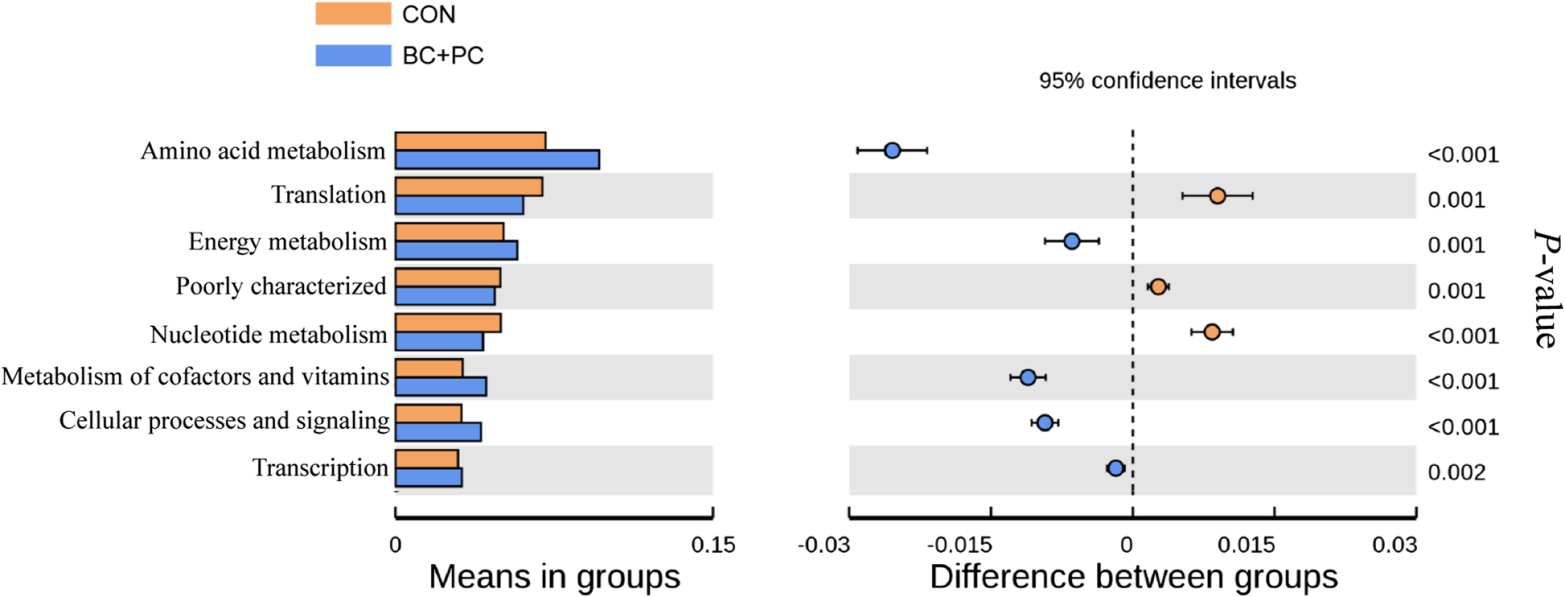
Prediction of the functional remodeling of ileal microbiota following BC + PC treatment *t*-test analysis of functional predictions for broiler chicken ileal microbiota

The changes in gut microbiota composition are highly correlated with gut health. Using PICRUSt, the predicted alterations in KEGG metabolic pathways of the microbial community in the BC + PC group significantly enhanced amino acid metabolism, energy metabolism, co-factor and vitamin metabolism, cellular processes and signaling, and transcriptional levels compared to the CON group (*P* < 0.001), as depicted in Fig. 7.

### PC masked the effects of BC + PC on the cecal microbiota in broiler chickens

**Fig. 8:**
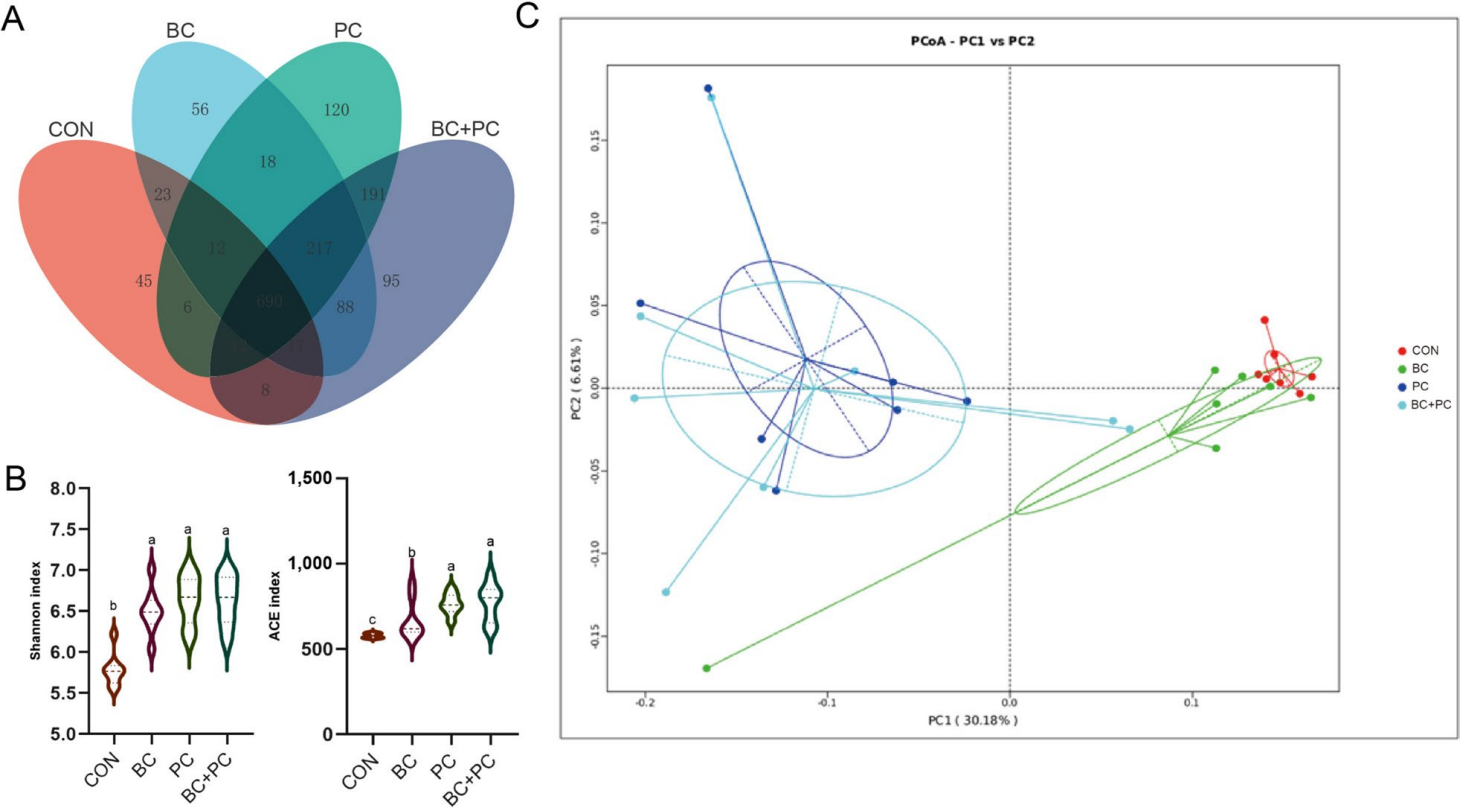
PC masked the effects of BC + PC on the cecal microbiota in broiler chickens. **A** The Venn diagram at the genus level. **B** Microbiome α-diversity was assessed at the genus level in BC, PC, BC + PC, and control groups (*n* = 8). **C** PCoA analysis was performed to evaluate the dissimilarity of the cecum microbial community structure (*n* = 8). ^a^^–^^c^Means with different superscripts within the same row are significantly different (*P* < 0.05)

To investigate the interactive effects of BC + PC on the cecal microbiota, α-diversity and β-diversity analyses were conducted. α-diversity analysis showed that the Shannon and ACE indices in the PC and BC + PC groups were not significantly different but significantly higher than the CON and BC groups (*P* < 0.05, Fig. 8B). Venn diagram analysis revealed 690 shared cecal microbiota OTUs across all treatment groups, with 45, 56, 120, and 95 unique OTUs in CON, BC, PC, and BC + PC groups, respectively(Fig. 8A). β-diversity analysis based on unweighted UniFrac distances exhibited distinct separation along PC2 axis (7.37%) and PC1 axis (17.61%), with CON and BC groups being close, and PC and BC + PC groups showing proximity(Fig. 8C).

**Fig. 9:**
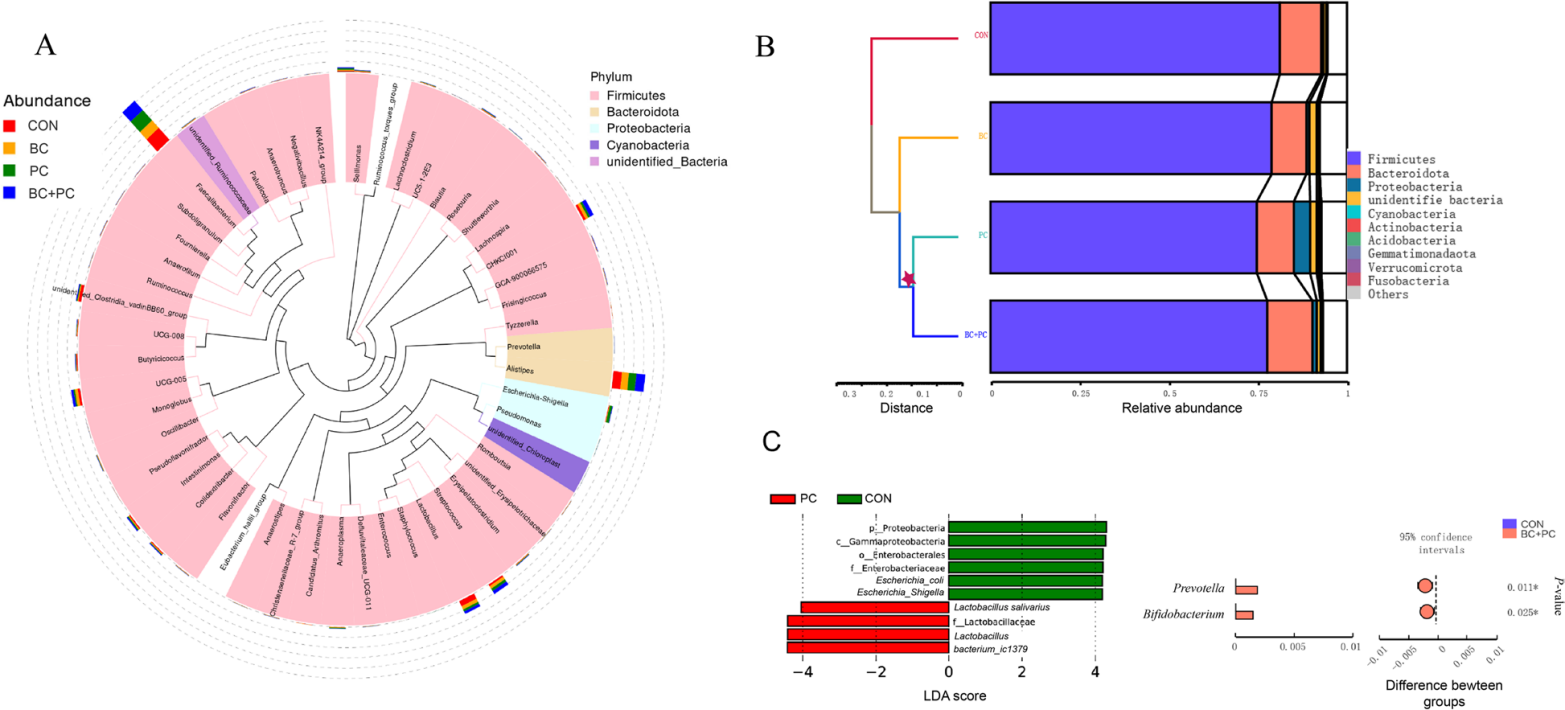
The effect of BC + PC combination on the cecal microbiota structure in broiler chickens. **A** Genus-level phylogenetic tree, where the inner circle represents genus-level bacteria, and the outer circle indicates their relative abundances. **B** UPGMA clustering analysis represents the similarity of microbial community composition based on distance. **C** LEfSe analysis was conducted on the CON, BC, PC, and BC + PC groups, with an LDA (Linear Discriminant Analysis) score cutoff of > 4. Additionally, a *t*-test was employed to examine CON and BC + PC, further identifying differentially abundant taxa

To assess the impact of different treatments on the cecal microbiota, we conducted genus-level phylogenetic trees and UPGMA clustering analysis (Fig. 9A). The results revealed a predominant presence of Firmicutes, followed by Bacteroidota in the cecal microbiota (Fig. 9A). UPGMA clustering analysis of the four treatment groups showed similar patterns to PCoA (Fig. 9B), indicating that PC exerted a similar effect to BC + PC in the broiler chicken intestine's end section. LEfSe analysis indicated that with LDA > 4, only CON and PC exhibited differences in certain microbial taxa, with Lactobacillaceae and *Lactobacillus* being dominant, further suggesting that PC masked the effects of BC in the cecal region (Fig. 9C). The genus-level *t*-test comparison between CON and BC + PC groups revealed *Prevotella* and *Bifidobacterium* as significantly different genera in the BC + PC group (*P* < 0.05, Fig. 9C).

### Correlation heatmap of gut microbiota, growth performance, intestinal immune and barrier-related parameters

**Fig. 10:**
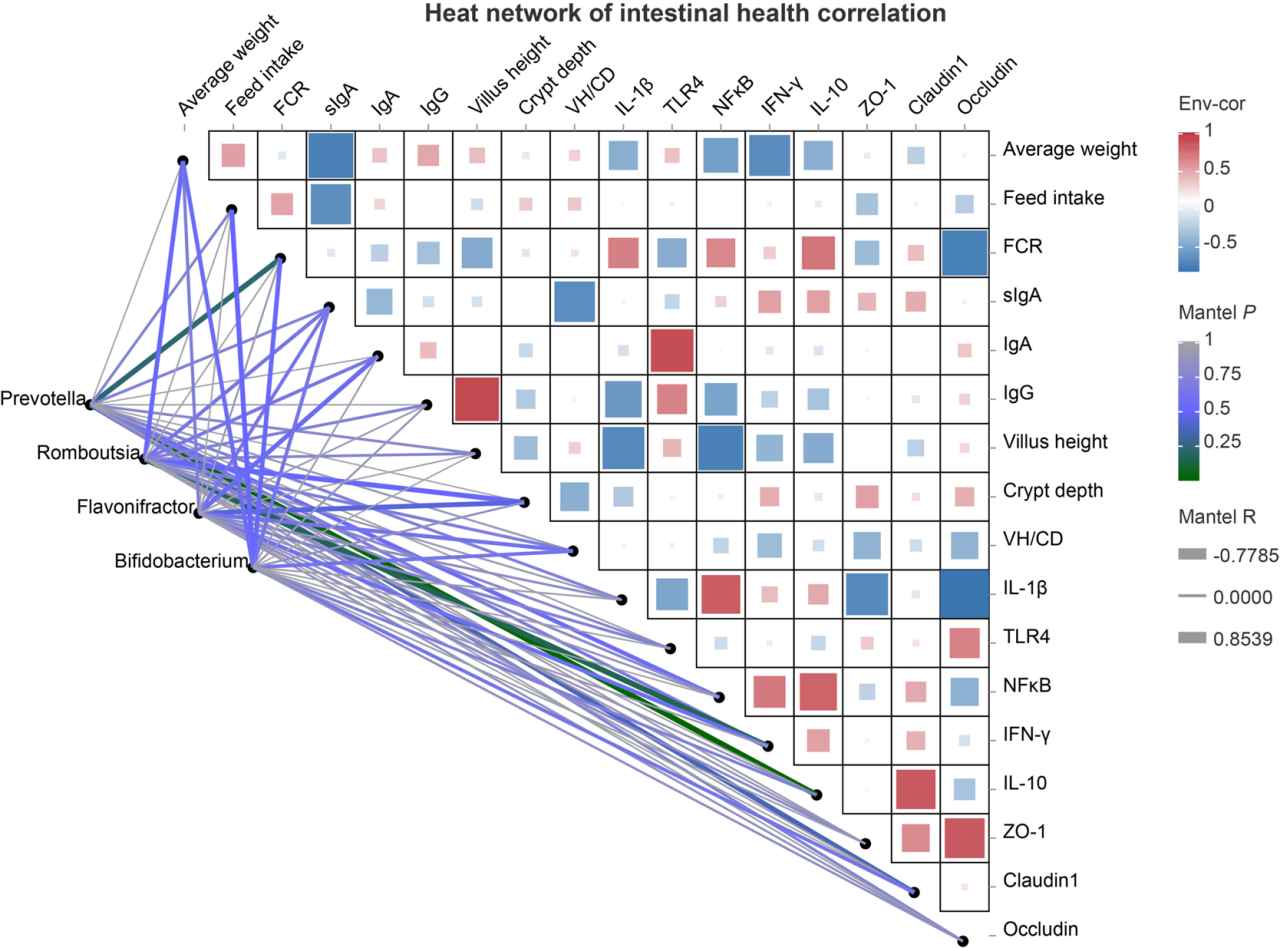
Correlation heatmap of gut microbiota, growth performance, intestinal immune and barrier-related parameters. The heatmap displays correlations between indicators and differentially abundant microbial taxa, with axes representing environmental factors and each cell showing correlation coefficients and magnitude. Connecting lines between differential microbial genera in the ileum and cecum relate to corresponding environmental factors, with line thickness and color indicating the strength and significance of the correlations

To investigate the correlation between differentially abundant genera in the ileum and cecum of the BC + PC group and broiler chicken production performance and gut health indicators, Spearman correlation analysis was performed (Fig. 10). Significant negative correlations were observed between sIgA, average weight, and feed intake (*P* < 0.05). Villus height showed significant negative correlations with *IL*-1*β* and *NF-κB* (*P* < 0.05). Within the ileal differential genera, *Romboutsia* demonstrated significant positive correlations with average weight and crypt depth (*P* < 0.05), while *Flavonifractor* showed significant positive correlations with IgA and crypt depth (*P* < 0.05). Moreover, among the cecal differential genera, both *Bifidobacterium* and *Prevotella* exhibited significant positive correlations with growth performance indicators (*P* < 0.05).

## Discussion

Plant based diets are rich in flavonoids. These compounds, together with their secondary metabolites, regulate gut and host health through microbial interactions [19]. Recent studies have indicated that dietary supplementation with flavonoids such as baicalin and its metabolite baicalein has no significant effect on broiler chicken's production performance [20, 21], which is consistent with our results. However, baicalin can improve serum antioxidant capacity under inflammatory conditions, reduce pro-inflammatory cytokine secretion, inhibit *NF-κB* gene expression, and alleviate intestinal inflammation and oxidative damage, thereby improving production performance [22]. This suggests that broiler chickens have a limited capacity to utilize baicalin under normal conditions, and its immunomodulatory effects may not be fully utilized. Our results show that the treatment with probiotic cocktails had no significant improvement in broiler chicken production performance, contrary to some existing reports [23]. This discrepancy may be attributed to variations in probiotic strains and their functional roles under different dietary conditions [24, 25], as well as variations in broiler response to probiotics [26]. BC and PC groups had no significant impact on the average body weight and feed intake from 1 to 42 d. However, they did enhance immune function, indicating that the dosage of both additives is appropriate, making them effective and beneficial feed additives. We observed that the BC + PC group significantly decreased the feed conversion rate from 21 to 42 d. Additionally, the BC + PC group showed a significant increase in sIgA level at 42 d, suggesting that BC + PC may induce a robust mucosal immune response at the expense of reduced feed conversion in chickens [27].

The intestinal villi are the primary site of nutrient absorption, and the integrity of the intestinal morphology has a significant impact on animal health. Increased villus height and width enhance the absorptive area, promoting nutrient uptake and optimizing production performance in broiler chickens [28]. Numerous studies indicate a strong correlation between gut health and host well-being, with inflamed chicken intestines infected by *Salmonella*, *E. coli*, coccidia, and LPS showing altered villus morphology and crypt depth [29–31]. Baicalin has been shown to alleviate these symptoms [32, 33]. Our research reveals that BC significantly reduces the crypt depth in the ileum of 21-day-old broiler chickens, suggesting a promoting effect on intestinal development under normal conditions. Studies indicate that flavonoids such as isoflavone and genistein can interact with the gut microbiota to increase the relative abundance of SCFA-producing bacteria, thereby promoting intestinal epithelial cell function and gut development [34]. Gut microbiota plays a critical role in this process, indicating that baicalin may also facilitate gut development through this mechanism [35, 36]. Interestingly, the combined treatment of BC + PC demonstrates a synergistic effect, significantly increasing the VH/CD ratio in the chicken ileum at 21 d, which may accelerate baicalin metabolism in the ileum and improve villus development through an interactive effect.

Recent studies have shown that BC and PC can improve the immune organ index of broiler chickens [14]. Our research demonstrates a synergistic effect of BC + PC treatment, which significantly increased the immune organ index of the spleen at 21 d and improved the immune organ index of the bursa of Fabricius and thymus at 42 d in broiler chickens. Immune factors play a critical role in the regulation of immune responses [36]. Serum levels of IgA and IgG are considered indicators of humoral immunity in poultry [37, 38]. sIgA is the primary defense of the intestinal immune barrier, inducing mucosal immune responses or immune tolerance and effectively clearing harmful antigens. Our results show that BC, PC, and BC + PC treatments did not promote the secretion of serum IgA and IgG under unchallenged conditions. However, BC and PC treatments enhanced the secretion of sIgA in the ileum of 42-day-old broiler chickens, which is consistent with previous studies showing that administration of propolis flavonoids to broiler chickens increased IgA levels in the ileum and cecum, thereby enhancing mucosal immune function [39]. *Lactobacillus reuteri* can manipulate Peyer's patches in the small intestine, utilize B cell plasticity and diversity, and promote sIgA induction and coating of gut microbiota [40]. Therefore, this selected combination of probiotics may improve immune function by modulating the humoral immune response of broiler chickens.

Cytokines are critical in regulating immune function and maintaining immune balance through the coordinated expression of pro-inflammatory and anti-inflammatory cytokines [41]. The TLR4 signaling pathway is a critical inflammatory pathway in the innate immune system and an essential target for the treatment of inflammation. Studies have shown that BC can inhibit CD14 expression, thereby suppressing TLR4/NF-κB p65 pathway activation and LPS-induced inflammatory responses [42]. It can also alleviate the inflammation-induced increases in *IL*-6 and *TNF-α* levels caused by *Salmonella enterica* [43] and treat *Escherichia coli*-induced acute lung injury in broiler chickens by regulating the NF-κB signaling pathway [44]. Our results indicate that BC, PC, and their combination did not affect *TLR*4 mRNA expression, possibly because the experiment did not induce inflammation. Thus, the immune function of the organism was not impaired, resulting in the absence of activation of the inflammatory signaling pathway and the absence of baicalin effects. Representative pro- and anti-inflammatory cytokines are *IL*-1*β* and *IL*-10 [45]. Studies have shown that baicalin can increase *IL*-1*β* expression by mediating *NLRP*3 inflammasome activation through the pyrin domain of *NLRP*3 [46]. It can also decrease *iNOS* and *TNF-α* expression by attenuating the stimulation of macrophages by pathogenic microorganisms [47]. In this study, the mRNA expression of *IL*-10 in the ileal mucosa of chickens treated with BC, PC, and BC + PC did not change significantly at 21 and 42 d. In contrast, the mRNA expression of *IL*-1*β* decreased significantly indicating an interaction effect. This suggests that under normal conditions, the complex polysaccharides of BC can reduce the activation of TLRs receptors and inhibit the transcriptional level of *IL*-1*β*, thereby improving the immune function of the organism. Further research is needed to fully understand this mechanism. Tight junction proteins are critical components of the intestinal epithelial barrier that protect the host from pathogen invasion. They consist of several types of proteins, including OCLN, claudins, and ZO-1. Baicalin can reduce intestinal permeability by increasing the mRNA expression of *ZO*-1 and *OCLN*, the tight junction proteins [48]. During intestinal inflammation, pro-inflammatory cytokines such as *IL*-1*β*, *TNF-α*, and *IFN-γ* are essential in regulating tight junction proteins [49]. Induced by *IL*-1*β*, *MAPK,* and *NF-κB* signaling pathways are activated, leading to a decrease in tight junction protein function and an increase in intestinal permeability [50, 51]. Our study indicates that BC, PC, and BC + PC treatment can decrease *IL*-1*β* mRNA expression in the ileum and increase the mRNA expression of occludin and claudin1, thereby improving intestinal health. Therefore, it can be inferred that the interactive effect of BC + PC may regulate the intestinal barrier by reducing the expression of pro-inflammatory cytokines.

The gut microbiota, which acts as the microbial barrier of the intestinal tract, is known to play a critical role in host metabolism, nutrition, physiology, and immune processes. *Lactobacillus*, found in the gut microbiota, tends to metabolize SCFAs and functional oligosaccharides, influencing the host's immune function [52]. However, harmful bacteria like *Escherichia coli* may produce endotoxins, affecting intestinal health [53]. Our study observed that BC and PC treatments altered the structure of the ileal microbiota, and increased relative abundance of species, with BC + PC showing a distinct β-diversity from BC and PC groups. This suggests a significant interaction between BC and PC in the chicken ileum. Studies indicate a close relationship between the efficiency of flavonoid metabolism and regulating host health [54]. Interestingly, in the chicken cecum, the influence of PC masked that of BC, as the structure of the PC microbiota resembled that of the BC + PC treatment, suggesting a potential acceleration of baicalin metabolism by probiotic cocktails, especially in the ileum.

In recent years, increasing research has emphasized the interaction between gut microbiota and plant extracts as a critical factor in pharmacological effects [55]. Studies indicate that in antibiotic-treated chicken models, baicalin (200 mg/t) exhibited a weakened preventive and therapeutic effect against *Escherichia coli*, suggesting that its action in poultry is mediated through gut microbiota metabolism [56]. Flavonoid transformation by gut microbiota can regulate intestinal health [57]. Recent findings show that *Flavonifractor* converts flavonoids into dihydrocaffeic acid (DAT), contributing to host protection against influenza [58]. *Flavonifractor* achieves this through flavonoid reductase (FLRs) catalyzing the hydrogenation of flavonoids [59, 60]. The study compared CON and BC + PC, BC and BC + PC groups, revealing significant differences in *Flavonifractor* abundance between BC and BC + PC groups, with BC + PC showing extremely significant differences compared to CON. This suggests that baicalin, as a substrate, promotes *Flavonifractor* growth, while the addition of probiotic cocktails facilitates this process, accelerating baicalin metabolism and enhancing absorption and efficacy [61]. Baicalin can be metabolized by the gut microbiota into highly active compounds, further enhancing its probiotic function. However, the specific mechanisms of baicalin metabolism and function in chickens remain to be explored.

Baicalin enhances gut health by increasing the abundance of short-chain fatty acid-producing bacteria such as *Blautia*, *Lachnospiraceae*, and *Intestinimonas* in the intestines [56]. SCFAs also act as prebiotics for beneficial microorganisms like *Bacillus subtilis*, *Lactobacillus plantarum*, and *Saccharomyces cerevisiae*, promoting their secretion [62–64]. The analysis conducted in this study, using LEfSe and *t*-test methods, revealed both BC and BC + PC treatments increased the relative abundance of the microbial communities compared to CON. The BC + PC group showed higher relative abundances of *Romboutsia*, *Prevotella*, and *Bifidobacterium*, with *Romboutsia* interacting synergistically with other potentially beneficial microorganisms. *Romboutsia* ferments various carbohydrates, producing SCFAs and oligosaccharides, contributing to the growth of different microbial communities and potential gut health benefits [65]. *Romboutsia* also plays a crucial role in host immunity [66]. Recent research indicates that supplementing with 50 g/t of soy isoflavones significantly increases *Romboutsia*'s relative abundance in the ileal chyme of laying hens, positively correlating with *IL*-4, *IFN-γ*, serum B lymphocyte secretion, and serum IgG levels [67]. In our study, *Romboutsia* showed a positive correlation with other potential beneficial microorganisms, sIgA, average weight, and crypt depth. *Prevotella*, as a cornerstone gut bacterium, significantly influences gut health, particularly in extracting propionate from arabinoxylan and oligofructose [68], possibly a crucial factor in promoting 21 d broiler intestinal development. *Bifidobacterium*, known to enhance gut health through the fructose 6-phosphate pathway, produces butyrate and acetate [69]. The result of the study demonstrates a significant positive correlation between *Bifidobacterium* and growth performance, further supporting improving gut health by regulating gut microbiota with BC + PC supplementation. Recent studies report that baicalin impacts nutrient metabolism, including energy, choline, and amino acid metabolism [70]. The KEGG enrichment analysis revealed that the combined use of BC + PC activated pathways related to amino acid metabolism, energy metabolism, co-factor, and vitamin metabolism, along with cell activity and signal transduction. These results indicate that the synergistic effect of BC + PC supplementation can improve the intestinal health of broilers, highlighting the significant interaction between the host microbiota and the major intestinal compartments.

## Conclusion

In conclusion, our study reveals a synergistic effect of combining baicalin and probiotic cocktails to significantly improve gut health in broilers. The combination of baicalin and probiotic cocktails significantly increases the relative abundance of *Flavonifractor*, a flavonoid-metabolizing bacterium, which enhances the metabolic efficiency of baicalin in the broiler ileum. This results in the production of small molecule compounds that regulate intestinal immunity, promote intestinal barrier function, and enhance nutrient absorption. Together, this combined approach contributes to the comprehensive regulation of broiler gut health, and opens novel perspectives for the efficient use of plant extract feed additives.

## Data Availability

The 16S rRNA gene sequencing data generated and analyzed during the current study are available in the NCBI primary data archive with accession number (PRJNA999083).

## Abbreviations

AA: Arbor Acres
AW: Average weight
BC: Baicalin
BWG: Body weight gain
CD: Crypt depth
CLDN-1: Claudin-1
DAT: Dihydrocaffeic acid
ELISA: Enzyme-linked immune sorbent assay
FCR: Feed conversion ratio
FLRs: Flavonoid reductase
IFN-γ: Interferon-γ
IgA: Immunoglobulin A
IgG: Immunoglobulin G
IL-1β: Interleukin-1β
IL-10: Interleukin 10
LDA: Linear Discriminant Analysis
LEfSe: Linear discriminant analysis combined effect size
NF-κB: Nuclear factor kappa-light-chain-enhancer of activated B cells
OCLN: Occludin
PC: Probiotic cocktails
PCoA: Principal coordinate analysis
PICRUSt: Phylogenetic investigation of communities by reconstruction of unobserved states
TLR4: Toll like receptor 4
VH: Villus height
VH/CD: Villus height to crypt depth ratio
ZO-1: Zonula occludens-1
